# Postoperative circulating tumor DNA can refine risk stratification in resectable lung cancer: results from a multicenter study

**DOI:** 10.1002/1878-0261.13387

**Published:** 2023-02-24

**Authors:** Rui Fu, Jun Huang, Xiaoru Tian, Chaoyang Liang, Yuanyuan Xiong, Jia‐Tao Zhang, Benyuan Jiang, Song Dong, Yuhua Gong, Wei Gao, Fang Li, Yonglei Shi, Zhentian Liu, Xuan Gao, Rongrong Chen, Wenzhao Zhong, Yi Zhang

**Affiliations:** ^1^ Guangdong Lung Cancer Institute, Guangdong Provincial Key Laboratory of Translational Medicine in Lung Cancer, Guangdong Provincial People's Hospital (Guangdong Academy of Medical Sciences) Southern Medical University Guangzhou China; ^2^ School of Medicine South China University of Technology Guangzhou China; ^3^ Department of Thoracic Surgery and Oncology, the First Affiliated Hospital of Guangzhou Medical University, State Key Laboratory of Respiratory Disease, National Clinical Research Center for Respiratory Disease Guangzhou Institute of Respiratory Health China; ^4^ Department of Thoracic Surgery Xuanwu Hospital, Capital Medical University Beijing China; ^5^ Department of Thoracic Surgery China‐Japan Friendship Hospital Beijing China; ^6^ Geneplus‐Beijing China

**Keywords:** ctDNA, molecular residual disease, non‐tissue‐derived ctDNA mutations, resectable lung cancer, risk stratification

## Abstract

Circulating tumor DNA (ctDNA) has potential as a promising biomarker for molecular residual disease (MRD) detection in lung cancer. As the next‐generation sequencing standardized panel for ctDNA detection emerges, its clinical utility needs to be validated. We prospectively recruited 184 resectable lung cancer patients from four medical centers. Serial postoperative ctDNAs were analyzed by a standardized panel. A total of 427 postoperative plasma samples from 177 eligible patients were enrolled. ctDNA positivity after surgery was an independent predictor for disease recurrence and preceded radiological recurrence by a median of 6.6 months (range, 0.7–27.0 months). ctDNA‐positive or ‐negative patients with tumors of any stage had similar disease‐free survival (DFS). Patients who received targeted therapy had significantly improved DFS than those not receiving adjuvant therapy or receiving chemotherapy, regardless of baseline/preadjuvant ctDNA status. According to whether the ctDNA variants were detected in its matched tissue, they were classified into tissue derived and non‐tissue derived. Patients with detectable postoperative ctDNA with tissue‐derived mutations had comparable DFS with those with non‐tissue‐derived mutations. Collectively, we demonstrated that postoperative ctDNA has the potential to stratify prognosis and optimize tumor stage in resectable lung cancer. ctDNA variants not identified in tissue samples should be considered in MRD test.

## Graphical abstract

Here, we examined the clinical utility of a standardized panel‐based circulating tumor DNA (ctDNA) test approach in 177 patients with resectable lung cancer. Our data demonstrated a significant correlation between postoperative ctDNA positivity and disease recurrence. Postoperative ctDNA positivity can further separate patients at high risk of recurrence in same tumor stage group. Postoperative ctDNA has the potential to stratify prognosis and optimize tumor stage in resectable lung cancer.

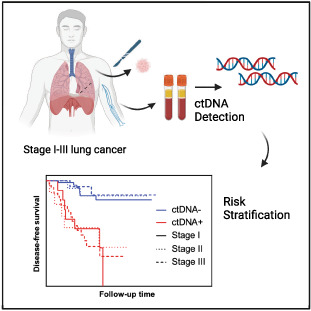

**Abbreviations**
CT: computed tomography
ctDNA: circulating tumor DNA
DFS: disease‐free survival
Indels: insertions and deletions
MRD: molecular residual disease
NGS: next‐generation sequencing
NPV: negative predictive value
NSCLC: non‐small cell lung cancer
OS: overall survival
SCLC: small cell lung cancer
SNV: single‐nucleotide variant
VAF: variant allele fraction

## 1 Introduction

Lung cancer remains the leading cause of cancer‐associated death worldwide, with over 1.8 million newly diagnosed cases and 1.59 million death cases per year [1]. Non‐small cell lung cancer (NSCLC) accounts for nearly 85% of these cases, and small cell lung cancer (SCLC) accounts for a majority of the remaining 15%. In approximately 30% patients with localized lung cancers, a subset can be cured after primary surgical resection, radiotherapy, and/or combined adjuvant therapy, while 8–64% of the patients experience recurrence [2], making them necessary for further therapy but poor outcomes. Systemic chemotherapy is the standard care for patients with stage IB NSCLC with high‐risk features, as well as patients with stage II–III NSCLC [3, 4]. However, the effect of adjuvant chemotherapy is modest, clinical studies have shown a 5‐year absolute benefit of 5.4% from adjuvant chemotherapy and no effectiveness even worsen outcomes in patients with stage IA NSCLC [5, 6]. For patients with completely resected *EGFR*‐mutant NSCLC, targeted therapy could significantly improve the disease‐free survival (DFS) compared with adjuvant chemotherapy, but some patients are refractory to targeted therapy and experience side effect of treatment [7, 8]. In addition, with the widespread implementation of lung cancer screening, the number of patients with resectable lung cancer will increase. Therefore, developing a tool to identify the patients who would benefit from adjuvant therapy is urgently needed and may improve the outcomes in the adjuvant therapy setting.

To reduce the threat of disease recurrence, clinical surveillance after curative‐intent therapies is recommended to detect recurrence early enough for applicable treatment. Radiographic imaging, including computed tomography (CT), magnetic resonance imaging, and positron‐emission tomography coupled with CT are commonly used for the surveillance. However, only macroscopic disease recurrence can be detected and clinical benefits are limited [9, 10, 11]. Therefore, there is a need to develop a sensitive and specific biomarker that can identify the patients at high risk of recurrence when the disease burden is minimal, and thereby applying adjuvant therapy.

Circulating tumor DNA (ctDNA) has emerged as a promising biomarker to predict molecular residual disease (MRD) after radical treatment in several cancers [12, 13, 14]. Recently, several ctDNA next‐generation sequencing (NGS) platforms have been developed to understand the performance of ctDNA in localized lung cancer, such as Natera, RaDaR™, and CAPP‐seq [15, 16, 17, 18]. The former two approaches use tumor‐informed and patient‐specific panel with applying whole‐exome sequencing on tumor, and the latter one is based on a tumor‐naïve and standardized panel targeting recurrent mutations in NSCLC. With no construction of a patient‐specific panel for each patient, a standardized panel makes for a far cheaper, faster, and resource‐intensive approach [19]. However, its clinical utility needs to be validated in more independent cohorts, especially in a multicenter study.

In this prospective multicenter study, we aimed to assess the utility of a standardized NGS panel on postoperative MRD detection for resected lung cancer patients.

## 2 Materials and methods

### 2.1 Patient recruitment and characteristics

This prospective, multicenter study recruited 184 Chinese patients with early‐stage lung cancer from February 16, 2017 to April 24, 2022, received radical surgery, which is an extensive surgery designed to remove all the tumor lesions, at Xuanwu Hospital, Capital Medical University, Guangdong Provincial People's Hospital, the First Affiliated Hospital of Guangzhou Medical University and China‐Japan Friendship Hospital. Notably, owing to insufficient awareness of potential benefits of MRD testing postoperatively during the first 2 years of our study (2017–2018), most patients were recruited in the years 2019–2022. Eligible patients were aged > 18 years, and with pathological stage I–III lung cancer, and no malignant tumor history within the past 5 years. The exclusion criteria are as follows: (a) multiple primary lung cancer, which is characterized by more than one cancerous lesion independent of each other; (b) R1, R2 resection. One hundred and seventy‐seven patients were eligible for analyzing the performance of postoperative ctDNA and their clinicopathologic data are summarized in Table 1. The classification of lung cancer and disease stage were defined by the World Health Organization criteria and the American Joint Committee on Cancer staging system, respectively. This study was approved by the Institutional Review Board of the Xuanwu Hospital, Capital Medical University (No. [2018]114) and Guangdong Provincial People's Hospital (No. GDREC2016175H[R2]). All patients provided written informed consent before enrollment. All research procedures conformed to the principles of the Helsinki Declaration.

**Table 1:** Demographic and baseline characteristics.

	*n* = 177
Age, years	
Mean (SD)	59.1 (10.1)
Median	60 (30–82)
Gender, *n* (%)	
Male	96 (54.2)
Female	81 (45.8)
Smoking, *n* (%)	
Never	87 (61.3)
Ever	55 (38.7)
Unknown	35
Tumor stage, *n* (%)	
0	1 (0.6)
I	89 (50.3)
II	28 (15.8)
III	59 (33.3)
Histology, *n* (%)	
Adenocarcinoma	149 (84.2)
Squamous cell carcinoma	19 (10.7)
Adenosquamous carcinoma	4 (2.2)
Large cell neuroendocrine carcinoma	2 (1.1)
Adenocarcinoma *in situ*	1 (0.6)
Sarcomatoid carcinoma	1 (0.6)
Small cell lung cancer	1 (0.6)
Lymph node metastasis, *n* (%)	
Yes	73 (41.2)
No	104 (58.8)
Recurrent disease, *n* (%)	
No	136 (76.8)
Yes	41 (23.2)
Follow‐up time, months	
Nonrecurrence, mean (SD)	18.2 (1.0–52.8)
Recurrence, mean (SD)	11.5 (1.4–27.3)

### 2.2 Sample collection

Tumor tissue was collected at surgery. Twenty milliliters of peripheral blood samples was collected in Streck tubes at preset time points after surgery. The landmark time point was defined as 1 month (±7 days) after surgery. Then, patients were scheduled to be followed every 3 or 6 months with CT scan and ctDNA test until disease recurrences determined by CT scan results. Longitudinal time point was defined as serial postoperative time points until disease recurrence (including disease recurrence time point).

### 2.3 Sample processing and DNA extraction

Tumor DNA was extracted from formalin‐fixed, paraffin‐embedded (FFPE) tumor tissue specimens using the ReliaPrep™ FFPE gDNA Miniprep System (Promega, Madison, WI, USA). Peripheral blood sample was separated by concentration at 1600 ***g*** for 10 min, and the supernatant was transferred to microcentrifuge tubes and centrifuged at 16 000 ***g*** for 10 min to remove cell debris. cfDNA was isolated using the QIAamp Circulating Nucleic Acid Kit (Qiagen, Hilden, Germany). Peripheral blood leukocytes (PBLs) from the first centrifugation step were collected to extract germline genomic DNA. To avoid the contamination of ctDNA, the junction between plasma and PBLs was firstly discarded, then PBLs were carefully transferred. QIAamp DNA Blood Mini Kit (Qiagen) was used to extract the germline genomic DNA from PBLs. The concentration and fragment distribution of cfDNA were assessed using an Agilent 2100 Bioanalyzer (Agilent Technologies, Inc., Santa Clara, CA, USA).

### 2.4 Targeted next generation sequencing

Circulating tumor DNA analyses were performed retrospectively by Geneplus Inc. (Beijing, China), with analysts blinded to patient outcome. Before library construction, 400–800 ng each of genomic DNA extracted from PBLs and tumor specimens was sheared into fragments at a 200–250 bp peak with a Covaris S2 ultrasonicator (Covaris, Woburn, MA, USA). A volume of 20–80 ng DNA from plasma was used for library construction, and unique identifiers were tagged on each double‐stranded DNA to distinguish authentic somatic mutations from artifacts. Indexed Illumina NGS libraries were prepared from PBL, tumor, and plasma DNA using the KAPA Library Preparation Kit (Kapa Biosystems, Wilmington, MA, USA).

DNA libraries of tumor and its paired germline were hybridized to a previously reported custom‐designed panel, which covers ~ 1.5 Mbp of the genome and targeting 1021 cancer‐related genes [19]. DNA libraries of plasma and its paired germline DNA were hybridized to a custom‐designed biotinylated oligonucleotide probe, covering 550 kbp of the genome and targeting 338 cancer‐related genes [19]. The hybridized libraries were sequenced using a 100‐bp paired‐end configuration on a DNBSEQ‐T7RS sequencer (MGI Tech, Shenzhen, China). The minimal mean effective depth of coverage for tissue/germline DNA and ctDNA was 300× and 3000× respectively.

### 2.5 Tumor somatic variant calling

After removal of terminal adaptor sequences and low‐quality reads with fastp [20], remaining reads were mapped to the reference human genome (hg19) and aligned using the burrows–wheeler aligner (version 0.7.12‐r1039) with default parameters. Duplicated reads were removed using markduplicates tool in picard (version 4.0.4.0; Broad Institute, Cambridge, MA, USA). realdcaller (v1.8.1; Geneplus‐Beijing, inhouse) [19, 21] and gatk (v3.6‐0‐g89b7209; Broad Institute) were employed to call tumor somatic single‐nucleotide variants (SNVs) and small insertions and deletions (indels). contra (2.0.8)  was used to identify copy number variations [22]. ncsv (v0.2.3; Geneplus‐Beijing, inhouse) was applied to detect structural variants [19, 23]. All candidate variants were manually confirmed with the integrative genomics viewer browser. Variants were filtered to exclude germline mutations in dbSNP, and those that occur at a population frequency of > 1% in ExAc (v0.3.1) or 1000 Genomes Project. Variants captured by PBLs sequencing that are canonically associated with hematologic malignancies, but do not meet the criteria for diagnosis of leukemia, are considered to have clonal hematopoiesis of indeterminate potential. An inhouse database of clonal hematopoiesis variants of > 10 000 pan‐cancer patients and healthy individuals was used to filter clonal hematopoiesis‐related variants [24].

### 2.6 ctDNA detection

The ctDNA detection method was reported previously [19] and detailed below. After removing duplicated reads and polishing sequencing errors using unique identifiers and realseq (v3.1.0; Geneplus‐Beijing, inhouse) [19, 21]. SNV and indels were called by using realdcaller and gatk. tnscope (Sentieon Inc., San Jose, CA, USA) was used as auxiliary software to improve the detection of long indels. Variants met following criteria were filtered out: (a) variants present in matched germline DNA; (b) variants occur at a population frequency of > 1% in ExAc (v0.3.1) or 1000 Genomes Project; (c) variants with positional depth < 300×, and (d) for sequencing error removal, a set of ~ 500 healthy plasma samples were sequenced to construct a sequencing background for each targeted SNV.

According to whether the plasma variants occurred in its matched tumor tissue, the resulting plasma variants were classified into tissue‐derived and ctDNA‐private. In other words, tissue‐derived ctDNA mutations also occurred in its matched tumor tissue, while ctDNA‐private did not. Tissue‐derived variants showed a statistically significant difference in background and were considered reliable. When the following conditions were met, there are considered to be true somatic mutations: for tissue‐specific driver mutations, ≥ 2 high‐quality support reads; and for other tissue‐specific nonrecurrent mutations, ≥ 4 high‐quality support reads. For ctDNA‐private variants, the reliable somatic mutations were identified if they met the following stringent conditions: (a) for hotspot mutations, ≥ 4 high‐quality support reads; (b) for nonhotspots, ≥ 8 high‐quality support reads; and (c) clonal hematopoiesis were filtered through deep sequencing of PBLs. Positive ctDNA was defined as at least one variant detected [19].

### 2.7 Statistical analysis

The primary outcome measure was DFS, assessed by standard radiologic criteria. DFS was defined as the time from the data of surgery to the first radiologic recurrence and was censored at last follow‐up. Overall survival (OS) was defined as the time from the data of surgery to death from any cause or last follow‐up. Statistical analyses were performed using spss version 26.0 (SPSS Company, Chicago, IL, USA) or r statistical software (version 4.1.2 for Windows). Survival analysis was performed using the Kaplan–Meier method and compared using log‐rank test. The Mann–Whiney *U* test and Student's *t*‐test were used for non‐normally and normally distributed continuous variables, respectively. Comparison of categorical variables was conducted with Pearson's χ^2^ test or Fisher's exact test. A two‐sided *P* value < 0.05 was considered significant. A multivariant analysis was performed using the cox proportional hazards regression analysis and including all the factors that were significant at univariate analysis.

## 3 Results

### 3.1 Description of the population

**Fig. 1:**
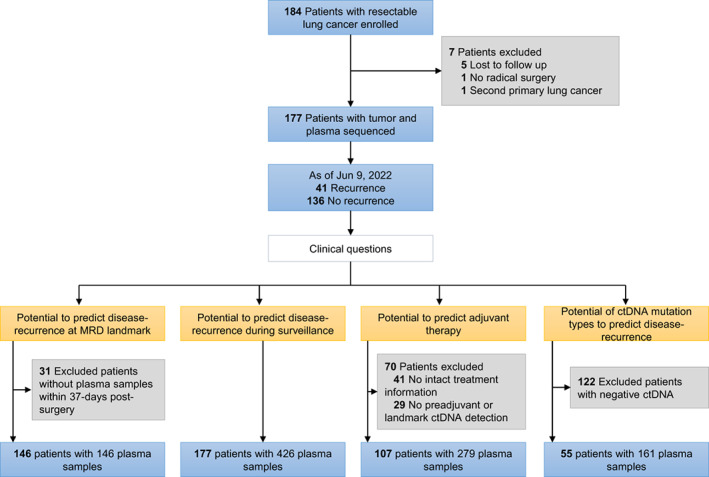
Patient enrollment and study overview. A total of 184 patients with resectable lung cancer were enrolled in this study. Five patients lost to follow‐up, and one without radical surgery and one with a second primary lung cancer were excluded. Finally, 177 patients were included in further analyses, including 146 with landmark plasma samples, 177 with postoperative longitudinal monitoring plasma samples, 107 with preadjuvant or landmark plasma samples, and 55 with detectable postoperative ctDNA.

In this study, a total of 184 patients with resectable lung cancer were enrolled. Five patients lost to follow‐up, one without R0 resection and one with second primary lung cancer were excluded (Fig. 1). Finally, 177 patients were included to analyze the performance of postoperative ctDNA, and their characteristics and demographics were listed in Table 1. The mean age at diagnosis was 59.1 years, with 54.2% males and 38.7% smokers. Most patients had stage I disease (50.3%, *n* = 89), followed by stage III (33.3%, *n* = 59) and II (15.8%, *n* = 28), with only one patient having adenocarcinoma *in situ*. Via clinicopathological examination, 84.2% (*n* = 149) patients had adenocarcinoma, with 10.7% (*n* = 19) squamous cell carcinoma, 2.2% (*n* = 4) adenosquamous carcinoma and 2.8% (*n* = 5) others. With a median follow‐up of 16.0 months (range, 1.0–52.8), disease relapse occurred in 41 patients (23.2%). Seven patients had died from disease relapse by the end of follow‐up period.

Tumor tissues were collected at surgery. Somatic mutations were detected by targeted NGS of tumor biopsies, and the mutation landscape of top 20 mutated genes of the 177 patients is shown in Fig. S1. A total of 1670 somatic mutations were identified, with a median of 6 (range, 1–48) gene variations detected in each patient. *EGFR* was the most common mutation and appeared in 100 patients (56.5%), followed by *TP53* (55.4%), *LRP1B* (16.9%), *KRAS* (13.6%), *RBM10* (11.9%), and *PIK3CA* (11.3%).

### 3.2 Association of postoperative ctDNA analysis with patient outcome

**Fig. 2:**
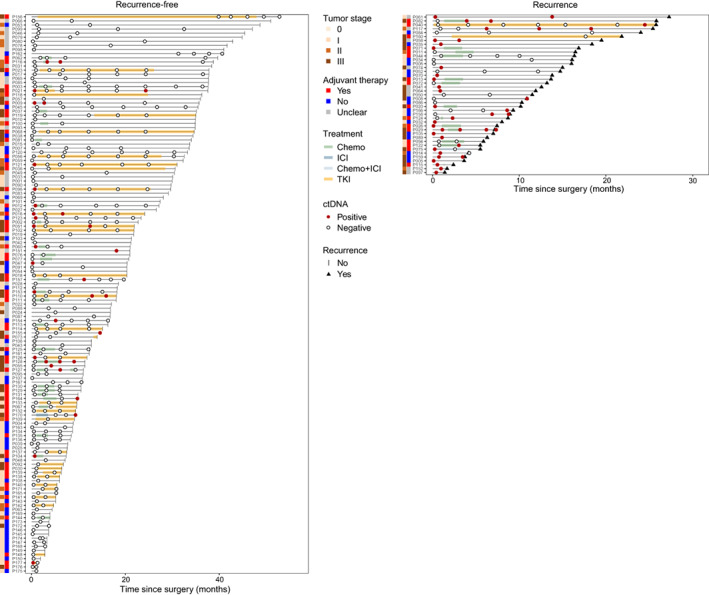
Longitudinal ctDNA monitoring and clinical course of patients in this study. Event chart showing clinical characteristics, DFS, and longitudinal ctDNA status in the overall cohort. Patients were separated by their radiographic outcome. Chemo, chemotherapy; ICI, immune checkpoint inhibitor; TKI, tyrosine kinase inhibitor.

A total of 427 postoperative blood samples were tested for ctDNA status, with an average 2.4 times for per patient (range, 1–8) (Fig. 2). A reported ultra‐deep targeted NGS panel covering 338 genes [19] was used to detect ctDNA (average effective sequencing depth of 4312×), tumor tissue mutations detected by a 1021‐gene panel [19] were used for classification of ctDNA mutation types.

**Fig. 3:**
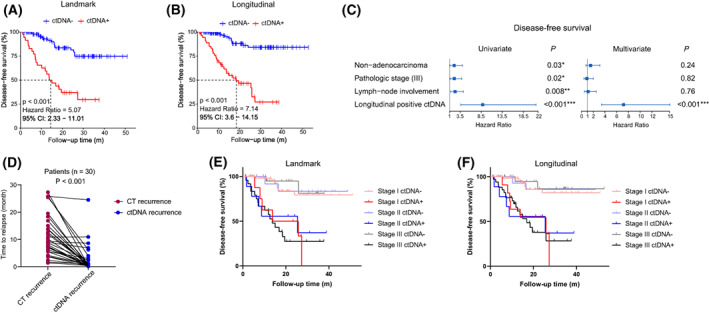
Disease‐free survival stratified by postoperative ctDNA status. (A, B) Kaplan–Meier estimates of DFS in patients stratified by ctDNA status at landmark (A) and during longitudinal surveillance (B). *P* value was calculated by the log‐rank test and hazard ratio by the Cox *exp*(*beta*) method. (C) Univariant and multivariate analysis for disease‐recurrence prediction with multiple clinicopathological variables and longitudinal positive ctDNA. (D) The comparison of the recurrence time measured by ctDNA versus CT. *P* value was calculated by two‐sided Wilcoxon two‐sample paired signed‐rank test. (E, F) DFS in different tumor stages stratified by ctDNA status at landmark (E) and during longitudinal surveillance (F). *P* value was calculated by the log‐rank test and hazard ratio by the Cox *exp*(*beta*) method.

A total of 146 patients were included for landmark ctDNA analysis. Among them, 36 patients (24.7%) were ctDNA positive at landmark time point, and 110 patients (75.3%) were negative. ctDNA positivity at landmark time point was associated with more advanced tumor stage and lymph node involvement before and after propensity score matching to balance baseline variables (Table S1). Landmark ctDNA‐positive patients had significantly higher recurrence rate (61.1% [22/36]) than those in ctDNA‐negative patients (14.5% [16/110]), producing sensitivity of 57.9% (22/38) and specificity of 87.0% (94/108). Moreover, the sensitivity was increased to 73.2% (30/41) and the specificity was 81.6% (111/136) in the longitudinal time point analysis. And the recurrence rate was also significantly higher in patient with longitudinal positive ctDNA (positive vs. negative, 54.5% [30/55] vs. 9.0% [11/122]) (Fig. 2). Consequently, patients with positive ctDNA at landmark or longitudinal time points had markedly reduced DFS (Fig. 3A,B). By performing univariate analysis, nonadenocarcinoma, advanced pathologic stage, lymph node involvement, and longitudinal positive ctDNA were associated with DFS, while longitudinal positive ctDNA was the only independent risk factor in the multivariant analysis (Fig. 3C). Additionally, median lead time from ctDNA detection to recurrence evaluated by standard‐of‐care CT was 6.6 months (range, 0.7–27.0 months) (Fig. 3D). It is worth noting that patients with tumors of any stage could be stratified by landmark or longitudinal ctDNA status, with ctDNA‐positive patients had significantly worse DFS than those in ctDNA‐negative patients. And ctDNA‐positive or ‐negative patients with tumors of any stage had similar DFS (Fig. 3E,F). We also compared OS among patients with positive versus negative ctDNA and found that patients with landmark or longitudinal positive ctDNA had significant shorter OS. However, owing to the large proportion of early‐stage disease and short follow‐up time, neither ctDNA‐positive nor ‐negative patients reached median OS at the time of data cutoff (Fig. S2A,B).

Previous study has reported that longitudinal undetectable ctDNA defines potentially cured population, which is supported by extremely high negative predictive value (NPV) [19]. We also assessed the NPV in our cohort, and found 88.4% of patients with longitudinal undetectable ctDNA were disease‐free at the end of follow‐up. The NPV was similar in different tumor stages, with 87.9% in stage I, 86.7% in stage II, and 90.5% in stage III (Fig. S3A). Eleven patients with negative postoperative ctDNA experienced disease recurrence. Of these, four patients (P086, P072, P006, and P011) had a time interval from last ctDNA detection to relapse more than 12 months. Patient P160 with stage IIIa adenocarcinoma experienced brain‐only metastasis, his negative ctDNA during targeted therapy was likely due to the low sensitivity of ctDNA for patients with brain‐only recurrence [19]. It has been extensively studied that ctDNA nonshedding of a tumor was associated with baseline clinicopathological features, such as adenocarcinoma histology and early tumor stage (stage I) [15, 16, 19, 25, 26]. For the remaining six patients, all of them harbored nonshedding clinicopathological features for ctDNA detection, with adenocarcinoma subtype (*n* = 1) or early tumor stage (Ib/Ic) (*n* = 2), or both (*n* = 3) (Fig. S3B). Additionally, to ascertain whether the false‐negative results were resulted from the incomplete referenced mutations due to the limited panel for tissue sequencing, we conducted whole‐exome sequencing on the tumor tissue of P052 and reanalyzed the corresponding ctDNA. As a result, the postoperative ctDNA of P052 remained negative after reanalyzing, suggesting that the undetectable ctDNA for P052 was not caused by incomplete referenced tissue mutations but probably the nonshedding clinicopathological features of tumor.

### 3.3 Association of adjuvant therapy with ctDNA clearance

**Fig. 4:**
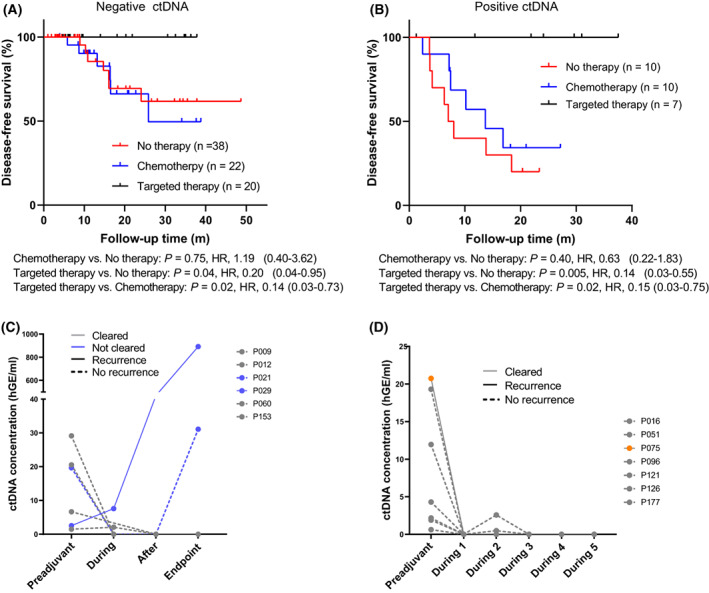
Association of DFS and adjuvant therapies in ctDNA‐positive and ‐negative patients. (A, B) Kaplan–Meier estimates of DFS in patients with undetectable (A) or detectable (B) landmark/preadjuvant ctDNA stratified by treatment strategies, including surgery only (no therapy), adjuvant chemotherapy, and adjuvant targeted therapy. *P* value was calculated by the log‐rank test and hazard ratio by the Cox *exp*(*beta*) method. (C) ctDNA level before, during, and after adjuvant therapy, and at time of recurrence or end of follow‐up (endpoint) for patients with detectable preadjuvant ctDNA. (D) ctDNA level before and during adjuvant therapy for patients with detectable preadjuvant ctDNA.

We then explored the association between adjuvant therapy and ctDNA status. Among 136 patients with intact treatment information, 59 patients received adjuvant chemotherapy (*n* = 32) or targeted therapy (*n* = 27) and had ctDNA test before adjuvant therapy as well as 48 patients who received surgery only and had landmark ctDNA test, were enrolled for this exploration. For the group with undetectable landmark or preadjuvant ctDNA, 7 out of 38 patients received surgery only experienced relapse, 6 out of 22 patients received chemotherapy experienced relapse, none of the patients received targeted therapy experienced relapse (0/20). Of those, comparable DFS was found between patients who received chemotherapy and surgery only, while longer DFS was found in patients who received targeted therapy when compared to the other two groups (Fig. 4A). However, it is worth noting that the baseline clinical features were uneven across these three groups, with patients who received surgery only tending to have early‐stage disease and patients who received targeted therapy tending to have a higher proportion of adenocarcinoma (Table S2). For the group with detectable landmark or preadjuvant ctDNA, patients who received targeted therapy had better DFS than those who received surgery only or chemotherapy (Fig. 4B). Most of landmark ctDNA‐positive patients (8/10) received surgery only and those who received chemotherapy (6/10) experienced relapse, while all patients who received targeted therapy (7/7) remained relapse‐free. Nevertheless, patients who received surgery only tended to be older (Table S3). In summary, patients who received adjuvant targeted therapy had better DFS and lower recurrence rate than those who received surgery only or adjuvant chemotherapy, regardless of preadjuvant/landmark ctDNA status. However, due to the limited sample size and uneven clinical characteristics across patients treated with different treatments, this result is preliminary.

Circulating tumor DNA level reflects tumor burdens [16, 27]. We then assess the impact of adjuvant therapy on ctDNA levels. Six preadjuvant ctDNA‐positive patients received ctDNA test during and after adjuvant therapy, of whom four were treated with chemotherapy, one with targeted therapy, and one with a combination of chemotherapy and immunotherapy. Five of them harbored a complete and permanent elimination of ctDNA at the end of adjuvant therapy and remained disease‐free, although one of them (P021) had regained positive ctDNA at the last follow‐up. One patient (P029) had continuously increased ctDNA levels during and after adjuvant therapy experienced recurrence (Fig. 4C). We also assessed the ctDNA levels for seven preadjuvant ctDNA‐positive patients who received ctDNA test before and during adjuvant therapy, of whom six received targeted therapy and one received a combination of chemotherapy and immunotherapy. Six of them presented cleared ctDNA levels during adjuvant and kept disease‐free, only one patient (P075) with cleared ctDNA during adjuvant therapy experienced relapse (Fig. 4D). Taken together, the dynamics of ctDNA status during or after adjuvant therapy can indicate the tumor response to therapy, and the clearance of ctDNA was associated with the clinical benefits from adjuvant therapy, while the persistent existence of ctDNA indicates the residual disease, which induces clinical recurrence.

### 3.4 Association of postoperative ctDNA mutation types with patient outcome

Considering a standardized panel makes for a commercially available MRD test approach, and tumor‐informed ctDNA analysis with having prior knowledge of tumor mutations could achieve maximal sensitivity for MRD test [28], our approach referred tissue mutations to stratify plasma variants captured by a standardized panel. According to whether the plasma variants occurred in its matched tissue, the resulting plasma variants were classified into tissue‐derived and ctDNA‐private. In other words, tissue‐derived ctDNA mutations also occurred in its matched tumor tissue, while ctDNA‐private did not. Compared with tissue‐derived mutations, ctDNA‐private mutations were filtered with a stricter rule (see Section 2).

**Fig. 5:**
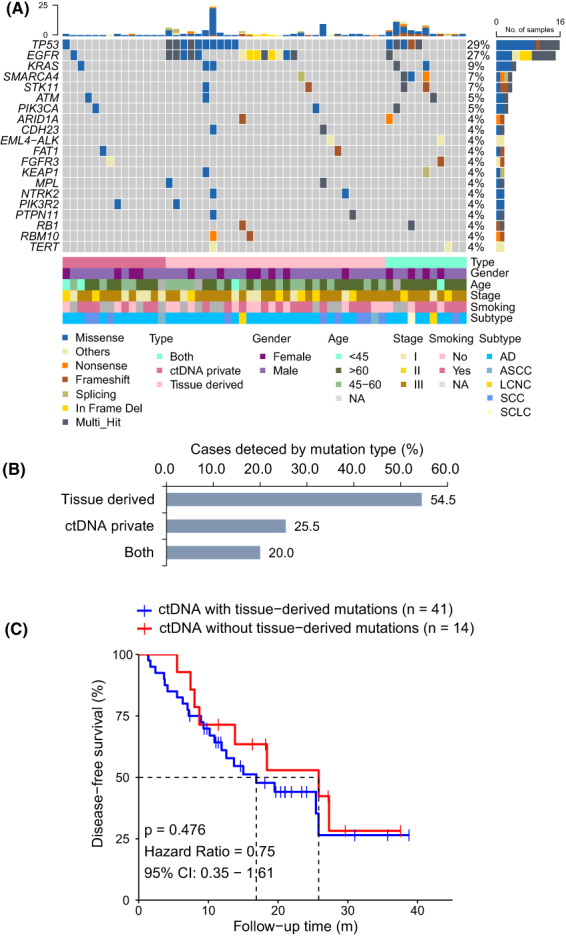
Association of postoperative ctDNA mutation types with patient outcome. (A) Mutational landscape based on postoperative detectable ctDNA analysis of 55 patients with resectable lung cancer. Each column represents data from a single patient. The number of somatic mutations of each patient and the mutation frequency of each gene are shown at the top and right, respectively. The bottom heatmaps indicate key patient characteristics. (B) The prevalence of different types of ctDNA mutations during postoperative surveillance was classified by whether the mutations were identified in its matched tissues. (C) Kaplan–Meier estimates of DFS in patients with or without tissue‐derived ctDNA mutations. *P* value was calculated by the log‐rank test and hazard ratio by the Cox *exp*(*beta*) method. AD, adenocarcinoma; ASCC, adenosquamous carcinoma; LCNC, large cell neuroendocrine carcinoma; SCC, squamous cell carcinoma.

The most common mutated genes in postoperative ctDNA were *TP53*, *EGFR*, *KRAS*, *SMARCA4*, and *STK11* (Fig. 5A). Of note, 54.5% (*n* = 30) patients harbored tissue‐derived mutations in their longitudinal plasma, 25.5% (*n* = 14) patients harbored ctDNA‐private mutations, and 20.0% (*n* = 11) patients had both tissue‐derived and ctDNA‐private mutations (Fig. 5B). During ctDNA surveillance, only five patients had mutation composition changes, of whom three had changes from ctDNA‐private mutation to tissue‐derived mutation, one had a change from tissue‐derived to ctDNA‐private mutation, and one had a change from both tissue‐derived and ctDNA‐private to tissue‐derived mutation. The median variant allele fraction (VAF) of tissue‐derived mutations was 0.31% (range, 0.01–20.26%), and the median VAF of ctDNA‐private mutations was 0.59% (range, 0.20–2.98%). However, despite the different filtering rules applied to these two kinds of ctDNA variants, no difference of VAF was found between them (*P* = 0.28). ctDNA‐private mutations were not caused by the limited coverage region of panel for tissue test, because the coverage region of our MRD‐test panel was entirely included in the tissue‐test panel. Thus, we investigated whether the differences of tissue sequencing condition and baseline molecular characteristics lead to the discrepancy of mutation types in their matched plasma. Our data showed that the percentage of tumor cells, effective sequencing depth, the number of somatic mutations, and the maximum somatic allele frequency were similar between the paired tumor tissues of plasma samples with different mutation types (Fig. S4). Furthermore, the mutational profiles in the paired tumor tissues of plasma samples with different mutation types were also similar (data not shown). Therefore, ctDNA variant types classified by whether it occurred in their paired tissues are not contributed from tissue's molecular features and sequencing condition. ctDNA‐private mutations are possibly generated from tumor heterogeneity, tumor evolution or acquired resistance to drug.

As ctDNA‐private mutations identified by our approach are ignored by patient‐specific panel with detecting tissue‐derived mutations approach, such as Natera and RaDaR™, we then investigated their utility for MRD test. Patients with tissue‐derived plasma mutations had similar clinical features with those with only ctDNA‐private mutations (Table S4). More importantly, there was no difference of recurrence rate (tissue‐derived vs. only ctDNA‐private: 53.7% [22/41] vs. 57.1% [8/14]) and DFS between them (Fig. 5C). This implies that ctDNA‐private mutations are also indispensable for ctDNA‐based MRD test.

## 4 Discussion

In this prospective, multicenter cohort study, we examined the clinical utility of a standardized panel‐based MRD test approach in 177 lung cancer patients treated with radical surgery with or without adjuvant therapy. Our data demonstrated a significant correlation between ctDNA positivity and disease recurrence. Postoperative ctDNA‐positive early‐stage lung cancer had a similar prognosis to that of locally advanced disease, and postoperative ctDNA‐negative stage III disease had a similar prognosis to that of early‐stage disease. Furthermore, by using our MRD‐test approach, we assessed the role of ctDNA mutations not detected in matched tumor tissue in risk‐stratification and found that both tissue‐derived and ctDNA‐private mutations are vital for MRD test.

The risk of recurrence after radical surgery in lung cancer has been traditionally estimated on the basis of tumor stage, which inform prognosis and the application of adjuvant therapy. However, because of the heterogeneity of patients, tumor stage is less accurate to recognize lung cancer patients with high‐ or low risk of recurrence and gives limited survival benefit from adjuvant therapy [5, 6, 29]. Our findings suggest that postoperative ctDNA test is a promising approach to predict recurrence possibility for patients with resectable lung cancer ahead of routine radiographic relapse. Especially, postoperative ctDNA positivity can further separate patients at high risk of recurrence in same tumor‐stage group, implying ctDNA status is a more elaborate disease‐recurrence predictor. According to this, we anticipate that except for tumor stage, postoperative ctDNA status should be considered in risk stratification. Indeed, a joint model combined postsurgical ctDNA status with other baseline clinical/pathological risk factors accurately predicts recurrence status in NSCLC [26]. Together, we propose that a “ctDNA‐based prognosis evaluation system” or a “ctDNA‐adjusted” TNM staging system in lung cancer warrants to be examined by prospective studies in the future.

Currently, tumor stage and clinical risk factors are used to select patients for adjuvant chemotherapy but with limited effectiveness. Adjuvant chemotherapy was found to confer a 5.4% 5‐year absolute benefit [5]. This modest survival benefit means that a quite subset of patients was cured by surgery. Based on previous studies, adjuvant chemotherapy could provide clinical benefit for postoperative ctDNA‐positive patients, but no effectiveness or even worsen outcome for landmark ctDNA‐negative patients [19, 25, 26]. However, these results were generated from chemotherapy or a mixed cohort consisting mainly of chemotherapy, little is known about the survival benefit of targeted therapy to patients with negative/positive preadjuvant ctDNA. In our study, no matter what landmark/preadjuvant ctDNA status is, patients received adjuvant targeted therapy had significantly longer DFS than patients received surgery only or adjuvant chemotherapy. Of note, our cohort was nonrandomized and small, which resulted in uneven clinical characteristics among patients who received targeted therapy, chemotherapy, and surgery only, further prospective, interventional large cohorts are needed to prove it. In addition, landmark/preadjuvant ctDNA‐negative patients who received chemotherapy had similar DFS to those who did not receive adjuvant therapy, which is consistent with previous studies [26]. From the survival curve, we observed a better DFS of patients who received chemotherapy than those who did not receive adjuvant therapy. The absence of statistical significance of DFS between these two groups may be explained by the limited number of patients enrolled in this analysis (chemotherapy, *n* = 10; no adjuvant therapy, *n* = 10). ctDNA status has been thought a promising biomarker to screen patients who would benefit from adjuvant therapy, and increasing ongoing studies research on whether ctDNA‐guided management is superior to standard management. Indeed, DYNAMIC study has demonstrated that ctDNA‐guided management could reduce the use of adjuvant chemotherapy without compromising recurrence risk in stage II colon cancer [30]. A prospective clinical trial (NCT05457049) is in progress to evaluate if patients with undetectable postoperative ctDNA are cured by surgery and do not need further adjuvant therapy. MERMAID‐1 study (NCT04385368) aims to assess the efficacy of durvalumab combined chemotherapy compared to placebo combined chemotherapy in terms of DFS measured in MRD‐positive patients.

For ctDNA detection, a standardized panel offers a cheaper, faster, and resource‐intensive approach compared to a patient‐specific panel. We performed a standardized panel targeting 338 genes which covers common mutations in lung cancer during ctDNA surveillance, and used tissue mutations captured by another standardized panel to classify ctDNA mutations. This approach has been evaluated by Zhang et al. [19] in 261 patients with stage I–III NSCLC who underwent radical surgery. The longitudinal ctDNA‐positive rate in our cohort was 31.1%, which is higher than that of Zhang's study (19.5%). More stage III disease in our cohort may explain this result (this study vs. Zhang's study: 33.3% vs. 17.2%). Longitudinal ctDNA detection produced a sensitivity of 73.2% and a specificity of 81.6% in our cohort. which is lower than Zhang's study (sensitivity: 87.2%, specificity: 97.4%). Divergence of median follow‐up time (this study vs. Zhang's study: 16.0 m vs. 19.7 m), clinical characteristics as well as treatment strategies (this study vs. Zhang's study; targeted therapy: 25.7% [35/136] vs. 11.5% [30/261]; chemotherapy: 27.2% [37/136] vs. 10.3% [27/261]; others: 3.7% [5/136] vs. 3.4% [9/261]) between these two studies may lead to this disparity.

Zhang et al. [19] reported that the preoperative ctDNA detection rate of this ctDNA detection method was 36.4% in the overall population, with 21.2%, 32.2%, 60.4%, and 48.9% positive rates of preoperative ctDNA in stage Ia, Ib, II, and III, respectively. Based on this, we estimated that the detection rate of preoperative ctDNA in our cohort was 40.2%, with 15.3%, 28.8%, 6.2%, 15.8%, and 33.3% patients in stage Ia, Ib, I, II, and III, respectively. Currently, diverse ctDNA sequencing platforms have appeared. Patient‐specific panel detecting tissue‐derived mutations and standardized NGS panel are two prevalent methods. For patient‐specific assays, such as RaDaR™ and Natera, the positivity of preoperative ctDNA in NSCLC ranges from 48% in Natera [16] to 51% in RaDaR™ [18]. For standardized NGS panel, such as CAPP‐seq, Zhang et al., Xia et al., Qiu et al., and Wang et al. [15, 19, 25, 26, 31], the positivity of preoperative ctDNA ranges from 20.9% in Xia et al. [25] to 93% in CAPP‐seq [15]. Due to the preoperative ctDNA positivity is largely dependent on clinical characteristics, head‐to‐head comparative trial is worthy to explore the clinical performance of patient‐specific and standardized NGS panel. Of great interest, several studies reported that some patients had undetectable preoperative ctDNA, their postoperative ctDNA could be detectable [16, 19, 25, 26]. For instance, in Zhang's study, among patients with longitudinal detectable ctDNA, 34.1% (14/41) had undetectable preoperative ctDNA [19]. Thus, the undetectable preoperative ctDNA may not indicate that ctDNA‐MRD monitoring is inappropriate. The limit of detection on VAF for MRD‐test also varies a lot among different assays. Patient‐specific assays have been developed to detect ctDNA down to a VAF of 0.002% or lower [9, 10]. The limit of detection on VAF in standardized NGS panel assays ranges from 0.5% in Wang et al. [7] and 0.002% in CAPP‐seq [2]. The detection limit largely depends on the breadth and depth of sequencing, which are negatively and positively correlated with the detection limit, respectively. Furthermore, the cost and time also increase with the increase in sequencing breadth and depth. Thus, assays with high detection sensitivity, low cost, and short time are more clinically applicable in MRD test setting.

Our result showed patients with ctDNA variants not occurring in matched tumor tissue had a similar clinical outcome with those with ctDNA variants found in their paired tissue. These ctDNA variants not identified in tissue sequencing are largely due to spatial tumor heterogeneity, tumor evolution, or acquired resistance to drug, which could not be captured by patient‐specific panels detecting tissue‐derived mutations or standardized panels referring tissue mutations only. Indeed, Jee et al. [32] reported that advanced NSCLC patients with ctDNA‐private mutations had worse OS than those with tissue‐matched ctDNA mutations, and these ctDNA‐private mutations featured subclonal drivers of resistance to targeted therapy. Ours and Jee's studies highlight the prognostic value of ctDNA mutations not matched in its tissue sample. Thus, we propose that ctDNA mutations not matched in its tissue sample are also vital for the MRD test. However, although plasma samples were prospectively collected in our study, ctDNA profiling was performed retrospectively. The clinical utility of ctDNA‐private mutations for the MRD test requires further studies.

There are potential limitations in our study. This multicenter prospective study had a limited number of patients. Larger studies are needed to further validate our findings, especially the clinical benefit of adjuvant targeted therapy in patients with detectable/undetectable preadjuvant ctDNA. Preoperative ctDNA detection was not performed in our cohort. Although a number of studies supported preoperative ctDNA appears to be a strong prognostic factor for clinical relapse in early‐stage lung cancer patients [17, 19, 25], little is known about the differences of recurrence rates and DFS in patients with nonshedding tumors and those with cleared ctDNA after surgery. Cohort with preoperative and surveillance ctDNA detection is warranted to explore it in the future.

## 5 Conclusions

In summary, we enrolled a multicenter cohort to investigate the performance of postoperative ctDNA in resectable lung cancer using a custom‐designed panel, and found postoperative ctDNA is a promising biomarker for refining risk stratification in resectable lung cancer. ctDNA‐private mutations should be taken into consideration in MRD test NGS panel design.

## Conflict of interest

The authors declare no conflict of interest.

## Author contributions

YZ, WZ, JH, and CL were involved in overall study design; RF, XT, J‐TZ, BJ, and SD were involved in collection and processing of clinical samples; RF, XT, J‐TZ, BJ, SD, YX, YG, WG, FL, YS, ZL, XG, and RC were involved in clinical data collection and curation; YX, YG, WG, ZL, and XG were involved in statistical and bioinformatics analyses; YZ, WZ, JH, CL, and RC were involved in supervised research; YX wrote first draft of paper; all authors reviewed and approved text.

### Peer review

The peer review history for this article is available at https://publons.com/publon/10.1002/1878‐0261.13387.

## Supporting information


**Fig. S1.** Mutational landscape of baseline tumor tissues.

**Fig. S2.** Overall survival stratified by postoperative ctDNA status.

**Fig. S3.** Analyses of postoperative ctDNA‐negative patients with disease relapse.

**Fig. S4.** The molecular characteristics and sequencing condition in the paired tumor tissues of plasma samples with different mutation types.

**Table S1.** Comparison of baseline clinical and molecular characteristics between patients with negative and with positive ctDNA at landmark.

**Table S2.** Comparison of baseline clinical characteristics between landmark/preadjuvant ctDNA‐negative patients with different treatments.

**Table S3.** Comparison of baseline clinical characteristics between landmark/preadjuvant ctDNA‐positive patients with different treatments.

**Table S4.** Comparison of baseline clinical and molecular characteristics between patients with different types of ctDNA.

## Data Availability

The data presented in this study are available on request from the corresponding authors. The data are not publicly available due to ethical restrictions.
