# Supplementary material for: Postoperative circulating tumor DNA can refine risk stratification in resectable lung cancer: results from a multicenter study
Source: Mol Oncol. 2023 Feb 24;17(5):825–38. doi: 10.1002/1878-0261.13387 (PMC10158775; doi:10.1002/1878-0261.13387)
Supplement: Supplementary file 1 — Fig. S1. Mutational landscape of baseline tumor tissues. [file MOL2-17-825-s005.pptx]

## Slide 1
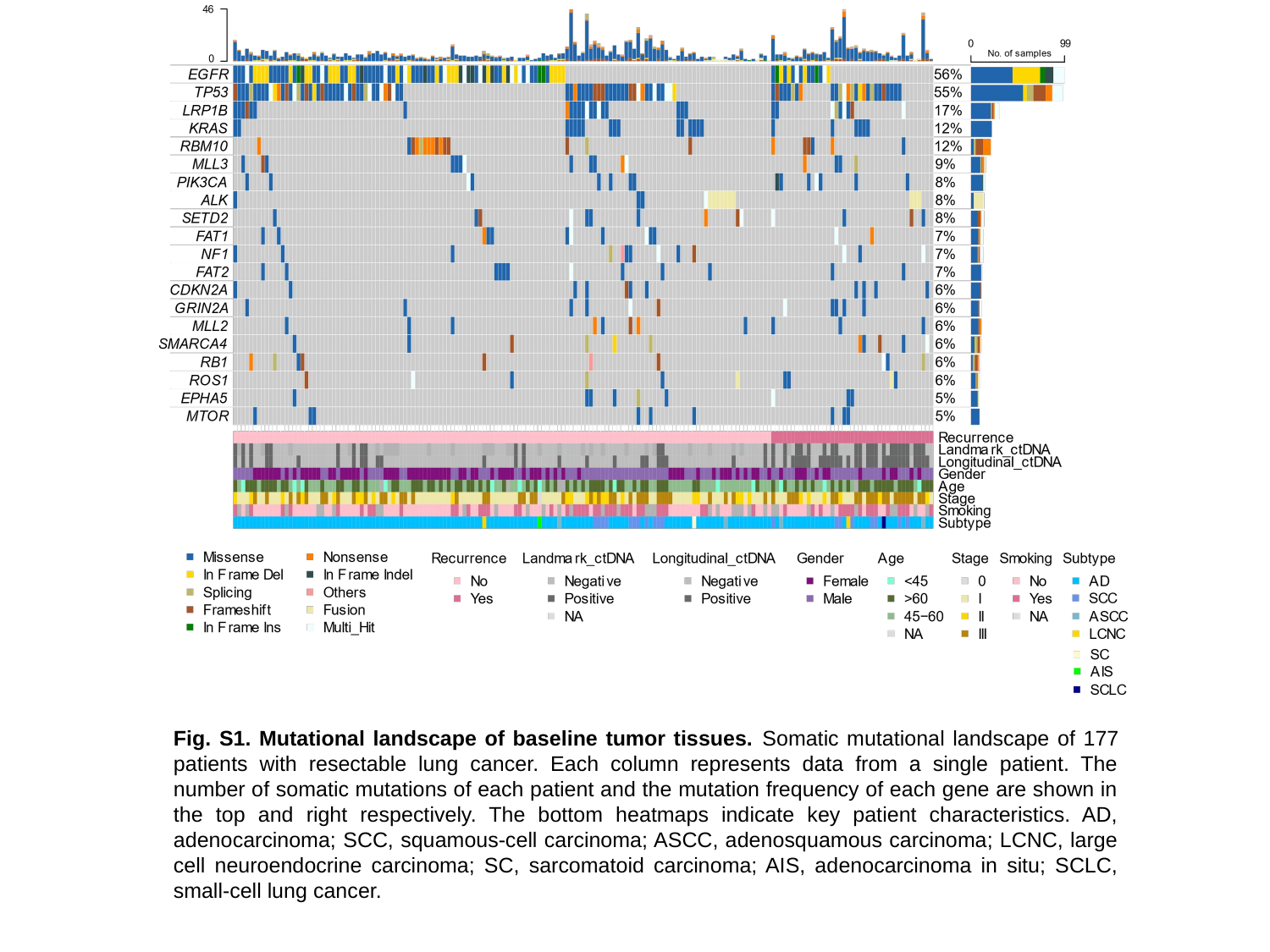

Fig. S1. Mutational landscape of baseline tumor tissues. Somatic mutational landscape of 177 patients with resectable lung cancer. Each column represents data from a single patient. The number of somatic mutations of each patient and the mutation frequency of each gene are shown in the top and right respectively. The bottom heatmaps indicate key patient characteristics. AD, adenocarcinoma; SCC, squamous-cell carcinoma; ASCC, adenosquamous carcinoma; LCNC, large cell neuroendocrine carcinoma; SC, sarcomatoid carcinoma; AIS, adenocarcinoma in situ; SCLC, small-cell lung cancer.
