# Supplementary material for: Postoperative circulating tumor DNA can refine risk stratification in resectable lung cancer: results from a multicenter study
Source: Mol Oncol. 2023 Feb 24;17(5):825–38. doi: 10.1002/1878-0261.13387 (PMC10158775; doi:10.1002/1878-0261.13387)
Supplement: Supplementary file 2 — Fig. S2. Overall survival stratified by postoperative ctDNA status. [file MOL2-17-825-s006.pptx]

## Slide 1
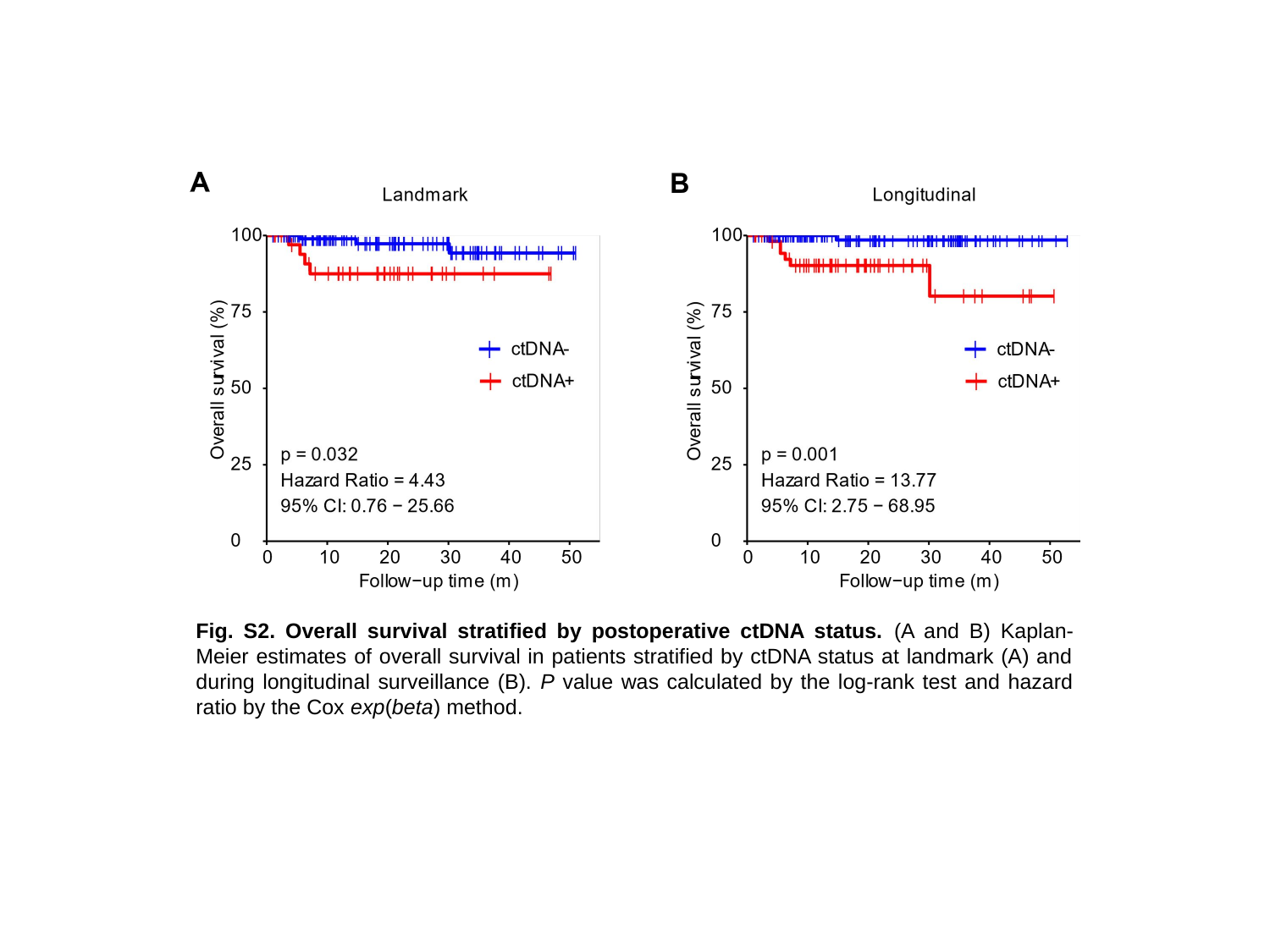

Fig. S2. Overall survival stratified by postoperative ctDNA status. (A and B) Kaplan-Meier estimates of overall survival in patients stratified by ctDNA status at landmark (A) and during longitudinal surveillance (B). P value was calculated by the log-rank test and hazard ratio by the Cox exp(beta) method.
