# Supplementary material for: Postoperative circulating tumor DNA can refine risk stratification in resectable lung cancer: results from a multicenter study
Source: Mol Oncol. 2023 Feb 24;17(5):825–38. doi: 10.1002/1878-0261.13387 (PMC10158775; doi:10.1002/1878-0261.13387)
Supplement: Supplementary file 3 — Fig. S3. Analyses of postoperative ctDNA‐negative patients with disease relapse. [file MOL2-17-825-s002.pptx]

## Slide 1
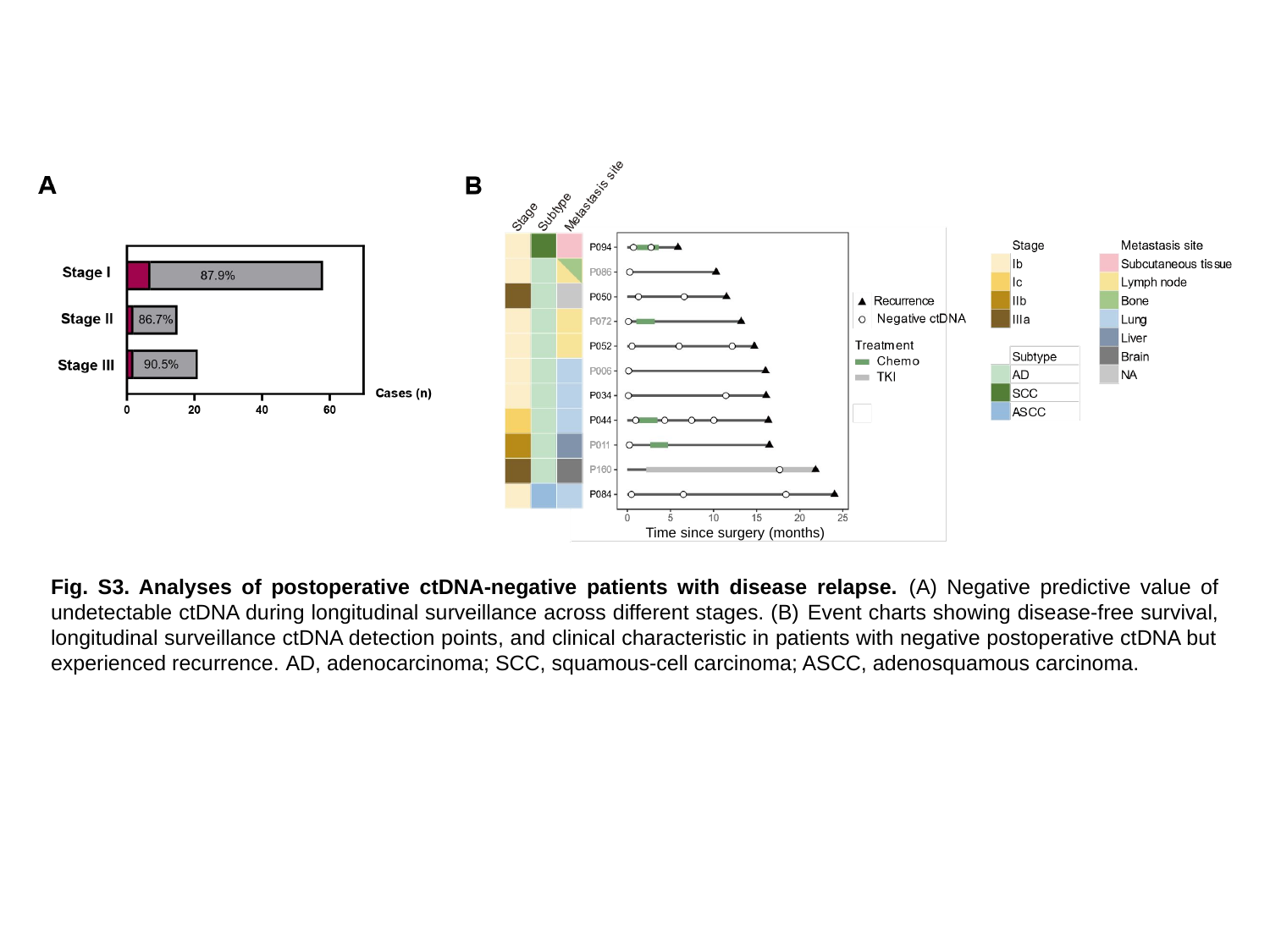

Time since surgery (months)
Fig. S3. Analyses of postoperative ctDNA-negative patients with disease relapse. (A) Negative predictive value of undetectable ctDNA during longitudinal surveillance across different stages. (B) Event charts showing disease-free survival, longitudinal surveillance ctDNA detection points, and clinical characteristic in patients with negative postoperative ctDNA but experienced recurrence. AD, adenocarcinoma; SCC, squamous-cell carcinoma; ASCC, adenosquamous carcinoma.
