# Supplementary material for: Postoperative circulating tumor DNA can refine risk stratification in resectable lung cancer: results from a multicenter study
Source: Mol Oncol. 2023 Feb 24;17(5):825–38. doi: 10.1002/1878-0261.13387 (PMC10158775; doi:10.1002/1878-0261.13387)
Supplement: Supplementary file 4 — Fig. S4. The molecular characteristics and sequencing condition in the paired tumor tissues of plasma samples with different mutation types. [file MOL2-17-825-s001.pptx]

## Slide 1
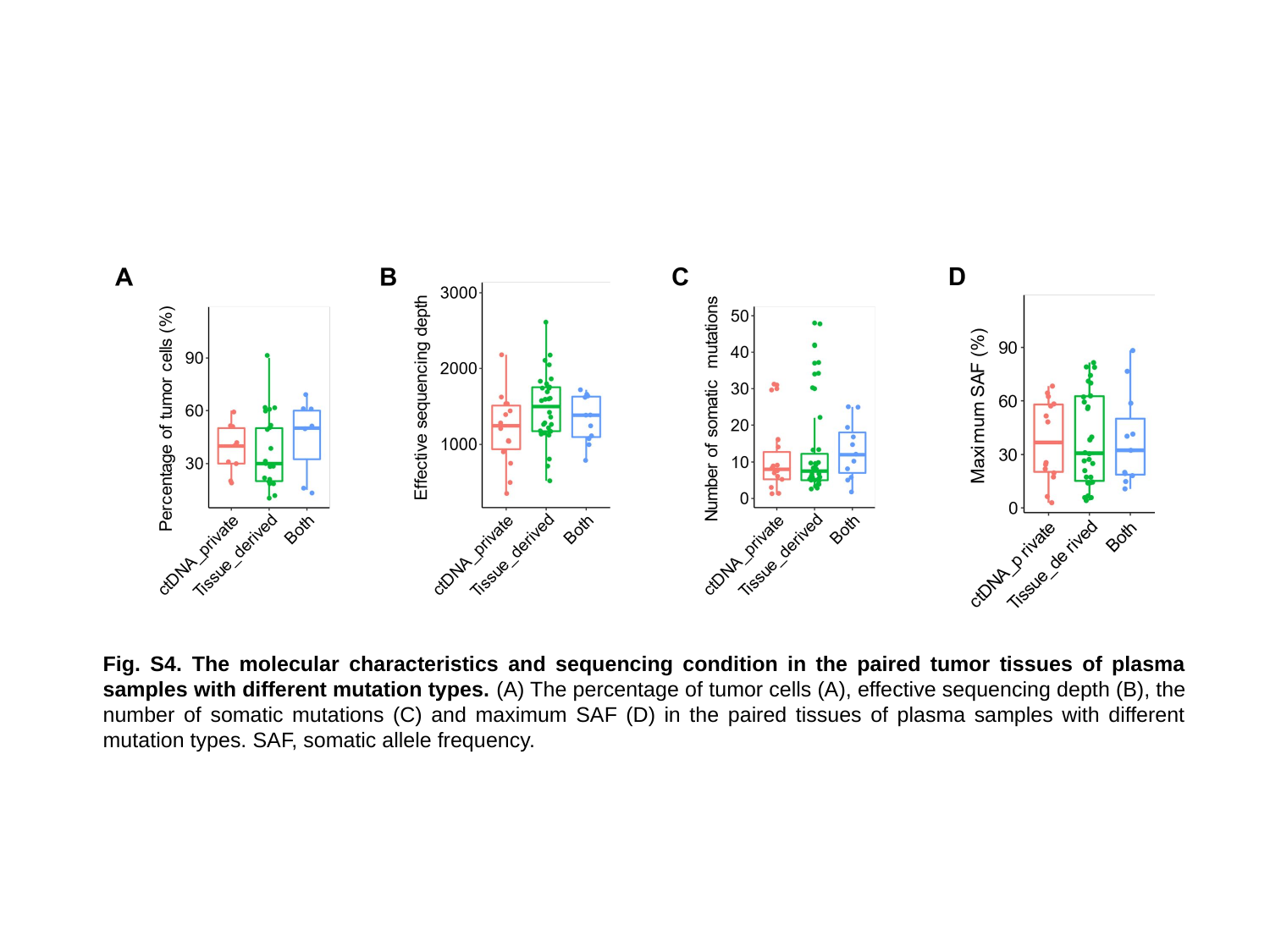

Fig. S4. The molecular characteristics and sequencing condition in the paired tumor tissues of plasma samples with different mutation types. (A) The percentage of tumor cells (A), effective sequencing depth (B), the number of somatic mutations (C) and maximum SAF (D) in the paired tissues of plasma samples with different mutation types. SAF, somatic allele frequency.
