# Supplementary material for: Postoperative circulating tumor DNA can refine risk stratification in resectable lung cancer: results from a multicenter study
Source: Mol Oncol. 2023 Feb 24;17(5):825–38. doi: 10.1002/1878-0261.13387 (PMC10158775; doi:10.1002/1878-0261.13387)
Supplement: Supplementary file 5 — Table S1. Comparison of baseline clinical and molecular characteristics between patients with negative and with positive ctDNA at landmark. [file MOL2-17-825-s003.docx]

**Table S1.** **Comparison of baseline clinical and molecular characteristics between patients with negative and with positive ctDNA at landmark.**

|  | **Positive (*n* = 36)** | **Negative (*n* = 110)** | ***P* value^a^** | **Negative after PSM (*n* = 36)** | ***P* value^b^** |
| --- | --- | --- | --- | --- | --- |
| **Age, years** |  |  | 0.42 |  | 0.76 |
| Mean (SD) | 60.5 (10.3) | 59.0 (9.7) |  | 59.9 (6.7) |  |
| Median | 62 (33-78) | 59 (35-82) |  | 61 (45-72) |  |
| **Gender, *n* (%)** |  |  | 0.18 |  | 0.63 |
| Male | 23 (63.9) | 55 (50.0) |  | 20 (55.6) |  |
| Female | 13 (36.1) | 55 (50.0) |  | 16 (44.4) |  |
| **Smoking, *n* (%)** |  |  | 0.13 |  | 0.79 |
| Never | 13 (46.4) | 58 (63.0) |  | 16 (53.3) |  |
| Ever | 15 (53.6) | 34 (37.0) |  | 14 (46.7) |  |
| **Tumor stage, *n* (%)** |  |  | <0.001^c^ |  | 0.004^c^ |
| I | 6 (16.7) | 66 (60.0) |  | 20 (55.6) |  |
| II | 6 (16.7) | 19 (17.3) |  | 5 (13.9) |  |
| III | 24 (66.6) | 25 (22.7) |  | 11 (30.5) |  |
| **Histology, *n* (%)** |  |  | 0.03^d^ |  | 0.4^d^ |
| Adenocarcinoma | 26 (72.2) | 98 (89.1) |  | 30 (83.3) |  |
| Squamous cell carcinoma | 8 (22.2) | 8 (7.3) |  | 4 (11.1) |  |
| Others | 2 (5.6) | 4 (3.6) |  | 2 (5.6) |  |
| **Lymph node metastasis, *n* (%)** |  |  | <0.001 |  | 0.002 |
| Yes | 26 (72.2) | 34 (30.9) |  | 12 (33.3) |  |
| No | 10 (27.8) | 76 (69.1) |  | 24 (66.7) |  |
| **TMB of baseline tumors** |  |  | 0.07 |  | 0.12 |
| Mean (SD) | 7.3 (7.2) | 4.8 (6.3) |  | 4.8 (4.8) |  |
| Median (min-max) | 5 (0-31) | 2.9 (0-46) |  | 3.8 (0-18) |  |

a Comparison between positive and negative ctDNA

b Comparison between positive and negative ctDNA after PSM

c Comparison between I-II and III

d Comparison between adenocarcinoma and non-adenocarcinoma

Abbreviation: PSM, propensity score matching; TMB, tumor mutation burden.
