# Supplementary material for: Postoperative circulating tumor DNA can refine risk stratification in resectable lung cancer: results from a multicenter study
Source: Mol Oncol. 2023 Feb 24;17(5):825–38. doi: 10.1002/1878-0261.13387 (PMC10158775; doi:10.1002/1878-0261.13387)
Supplement: Supplementary file 6 — Table S2. Comparison of baseline clinical characteristics between landmark/preadjuvant ctDNA‐negative patients with different treatments. [file MOL2-17-825-s004.docx]

**Table S2. Comparison of baseline clinical characteristics between landmark/preadjuvant ctDNA-negative patients with different treatments.**

|  | **Landmark/preadjuvant negative ctDNA** | | | | | |
| --- | --- | --- | --- | --- | --- | --- |
|  | **No therapy (*n* = 38)** | **Chemo (*n* = 22)** | **TKI (*n* = 20)** | **No therapy vs. Chemo (*P*val)** | **No therapy vs. TKI (*P*val)** | **Chemo vs. TKI (*P*val)** |
| **Age, years** |  |  |  | 0.94 | 0.77 | 0.72 |
| Mean (SD) | 59.4 (10.6) | 59.6 (8.1) | 58.5 (12.2) |  |  |  |
| Median | 59 (35-82) | 59 (45-74) | 60.5 (35-79) |  |  |  |
| **Gender, *n* (%)** |  |  |  | 0.11 | 0.78 | 0.35 |
| Male | 15 (39.5) | 14 (63.6) | 9 (45.0) |  |  |  |
| Female | 23 (60.5) | 8 (36.4) | 11 (55.0) |  |  |  |
| **Smoking, *n* (%)** |  |  |  | 0.24 | 1.00 | 0.33 |
| Ever | 9 (23.7) | 9 (41.0) | 5 (25.0) |  |  |  |
| Never | 26 (68.4) | 12 (54.5) | 14 (70.0) |  |  |  |
| Unknown | 3 (7.9) | 1 (4.5) | 1 (5.0) |  |  |  |
| **Tumor stage, *n* (%)** |  |  |  | 0.008 | 0.003 | 0.76 |
| I | 31 (81.6) | 10 (45.5) | 8 (40.0) |  |  |  |
| II-III | 7 (18.4) | 12 (54.5) | 12 (60.0) |  |  |  |
| **Histology, *n* (%)** |  |  |  | 0.15 | 0.29 | 0.02 |
| Adenocarcinoma | 34 (89.5) | 16 (72.7) | 20 (100) |  |  |  |
| Non-adenocarcinoma | 4 (10.5) | 6 (27.3) | 0 |  |  |  |

Abbreviation: Chemo, chemotherapy; TKI, tyrosine kinase inhibitor; *P*val, *P* value.
