# Supplementary material for: Postoperative circulating tumor DNA can refine risk stratification in resectable lung cancer: results from a multicenter study
Source: Mol Oncol. 2023 Feb 24;17(5):825–38. doi: 10.1002/1878-0261.13387 (PMC10158775; doi:10.1002/1878-0261.13387)
Supplement: Supplementary file 7 — Table S3. Comparison of baseline clinical characteristics between landmark/preadjuvant ctDNA‐positive patients with different treatments. [file MOL2-17-825-s007.docx]

**Table S3. Comparison of baseline clinical characteristics between landmark/preadjuvant ctDNA-positive patients with different treatments.**

|  | **Landmark/preadjuvant positive ctDNA** | | | | | |
| --- | --- | --- | --- | --- | --- | --- |
|  | **No therapy (*n* = 10)** | **Chemo (*n* = 10)** | **TKI (*n* = 7)** | **No therapy vs. Chemo (*P*val)** | **No therapy vs. TKI (*P*val)** | **Chemo vs. TKI (*P*val)** |
| **Age, years** |  |  |  | 0.07 | 0.06 | 0.81 |
| Mean (SD) | 67.0 (7.1) | 58.8 (11.3) | 58.0 (10.1) |  |  |  |
| Median | 66 (56-78) | 61 (33-75) | 61 (42-72) |  |  |  |
| **Gender, *n* (%)** |  |  |  | 0.63 | 1.00 | 0.59 |
| Male | 6 (60.0) | 8 (80.0) | 4 (57.1) |  |  |  |
| Female | 4 (40.0) | 2 (20.0) | 3 (42.9) |  |  |  |
| **Smoking, *n* (%)** |  |  |  | 1.00 | 0.59 | 1.00 |
| Ever | 4 (40.0) | 5 (50.0) | 3 (42.9) |  |  |  |
| Never | 2 (20.0) | 4 (40.0) | 4 (57.1) |  |  |  |
| Unknown | 4 (40.0) | 1 (10.0) | 0 |  |  |  |
| **Tumor stage, *n* (%)** |  |  |  | 0.58 | 0.60 | 1.00 |
| I | 3 (30.0) | 1 (10.0) | 1 (15.3) |  |  |  |
| II-III | 7 (70.0) | 9 (90.0) | 6 (85.7) |  |  |  |
| **Histology, *n* (%)** |  |  |  | 1.00 | 0.25 | 0.10 |
| Adenocarcinoma | 7 (70.0) | 6 (60.0) | 7 (100) |  |  |  |
| Non-adenocarcinoma | 3 (30.0) | 4 (40.0) | 0 |  |  |  |

Abbreviation: Chemo, chemotherapy; TKI, tyrosine kinase inhibitor; *P*val, *P* value.
