# Supplementary material for: Postoperative circulating tumor DNA can refine risk stratification in resectable lung cancer: results from a multicenter study
Source: Mol Oncol. 2023 Feb 24;17(5):825–38. doi: 10.1002/1878-0261.13387 (PMC10158775; doi:10.1002/1878-0261.13387)
Supplement: Supplementary file 8 — Table S4. Comparison of baseline clinical and molecular characteristics between patients with different types of ctDNA. [file MOL2-17-825-s008.docx]

**Table S4. Comparison of baseline clinical and molecular characteristics between patients with different types of ctDNA.**

|  | **ctDNA with tissue-derived mutation (*n* = 30)** | **ctDNA with private mutation (*n* = 14)** | **Both (*n* = 11)** | ***P* value^a^** |
| --- | --- | --- | --- | --- |
| **Age, years** |  |  |  | 0.41 |
| Mean (SD) | 58.5 (8.5) | 54.5 (11.8) | 66.9 (9.1) |  |
| Median (min-max) | 60 (42-75) | 62 (33-76) | 69.5 (44-78) |  |
| **Gender, *n* (%)** |  |  |  | 1.00 |
| Male | 23 (76.7) | 10 (71.4) | 7 (63.6) |  |
| Female | 7 (23.3) | 4 (28.6) | 4 (36.4) |  |
| **Smoking, *n* (%)** |  |  |  | 0.71 |
| Never | 11 (47.8) | 3 (33.3) | 3 (33.3) |  |
| Ever | 12 (52.2) | 6 (66.7) | 6 (66.7) |  |
| Unknown | 7 | 5 | 2 |  |
| **Tumor stage, *n* (%)** |  |  |  | 0.35^b^ |
| I | 6 (20.0) | 5 (35.7) | 1 (9.1) |  |
| II | 5 (16.7) | 2 (14.3) | 2 (18.2) |  |
| III | 19 (63.3) | 7 (50.0) | 8 (72.7) |  |
| **Lymph node metastasis, *n* (%)** |  |  |  | 0.09 |
| Yes | 23 (76.7) | 7 (50.0) | 9 (81.8) |  |
| No | 7 (23.3) | 7 (50.0) | 2 (18.2) |  |
| **Histology, *n* (%)** |  |  |  | 1.0^c^ |
| Adenocarcinoma | 22 (73.3) | 10 (71.4) | 8 (72.7) |  |
| Squamous cell carcinoma | 6 (20.0) | 3 (21.4) | 1 (9.1) |  |
| Others | 2 (6.7) | 1 (7.2) | 2 (18.2) |  |
| **Metastatic sites, *n* (%)** |  |  |  | 0.51 |
| Intrathoracic | 5 (45.5) | 0 | 0 |  |
| Extrathoracic | 6 (54.5) | 3 (100) | 3 (100) |  |
| Unknown | 3 | 5 | 5 |  |
| **TMB of baseline tumors** |  |  |  | 0.66 |
| Mean (SD) | 5.9 (6.5) | 7.3 (6.2) | 8.9 (5.7) |  |
| Median (min-max) | 3.8 (0-31.0) | 5.4 (1.0-21.0) | 8 (1.0-22.1) |  |

a Comparison between ctDNA with (ctDNA with tissue-derived mutation plus both) and without tissue-derived mutations (ctDNA with private mutation)

b Comparison between stage I-II and stage III

c Comparison between adenocarcinoma and non-adenocarcinoma

Abbreviation: TMB, tumor mutation burden.
